# Reply to the comment on the “Association between the Kidney Donor Profile Index and one-year outcomes in Brazilian kidney transplant recipients of standard criteria donors”

**DOI:** 10.1590/2175-8239-JBN-2025-0255rpen

**Published:** 2025-11-28

**Authors:** Ana Paula Aquino de Morais, Renato Demarchi Foresto, Maria Amélia Aguiar Hazin, Bianca Cristina Cassão, Helio Tedesco-Silva, José Medina-Pestana, Lúcio Requião-Moura

**Affiliations:** 1 Universidade Federal de São Paulo, Departamento de Medicina, São Paulo, SP, Brazil.; 2 Fundação Oswaldo Ramos, Hospital do Rim, São Paulo, SP, Brazil.

We would like to thank Parveen and Abid for their careful reading and thoughtful comments on our article, which evaluated the Kidney Donor Profile Index (KDPI) in Brazilian kidney transplant recipients (KTRs) from standard criteria donors^1^. Their observations raise relevant methodological and clinical considerations that contribute to the ongoing discussion of donor risk assessment.

We recognize that long-term graft survival is a more robust hard outcome compared to one-year estimated glomerular filtration rate (eGFR) in KTRs. However, as our study was an initial step in validating the KDPI for Brazilian donors, one-year follow-up was the most affordable and feasible approach at this stage. Nonetheless, it should not be ignored that one-year graft function or even eGFR slope has been previously associated with long-term outcomes^2^ and used as a surrogate endpoint in prior validation studies^3^. Still, a satisfactory one-year eGFR does not guarantee sustained graft function, as patient adherence to immunosuppressive therapy remains a well-recognized challenge for transplant teams. The ADHERE Brasil multicenter study reported a nonadherence prevalence of 39.7% in Brazilian KTRs^4^. Poor adherence is associated with chronic graft injury and graft loss through mechanisms such as high variability in tacrolimus trough levels and formation of de novo donor-specific antibodies, which may not be fully manifested within the first year^5^. To address these limitations, longer-term follow-up analyses of our cohort are underway and will be published to provide more comprehensive data.

Regarding statistical modeling, we agree that including clinically relevant variables, irrespective of their univariate significance, is a valid approach. Also, alternative strategies, such as predefining confounders based on clinical relevance, may strengthen external validity. In our analysis, we applied a conventional criterion of p < 0.10 in univariate testing to minimize the risk of overfitting the model, while ensuring that key clinically relevant variables were included. These variables showed significant and independent associations with the primary outcome, thereby supporting data robustness.

During most of the study period (2013–2017), pp65 antigenemia was the standard method for CMV monitoring in our center, with PCR testing implemented later after mid-2017^6^. This retrospective cohort analysis focused on graft function and graft survival as primary outcomes, with CMV-related events included as variables for adjustment in the analyses. Nakamura et al.^6^ further analyzed the transition period from antigenemia to RT-PCR testing in our center, and found that neither the incidence of CMV-related events nor the time to diagnosis differed between the two methods, except for a longer treatment duration with the PCR-based preemptive strategy.

In conclusion, despite the inherent limitations of a retrospective single-center cohort study, our findings demonstrate that a high KDPI is independently associated with poor outcomes, even among SCD transplants, and reinforce the usefulness of KDPI as a donor risk assessment tool in our context.

## Data Availability

No new data were created or analyzed in this manuscript.

## References

[1] de Morais APA, Foresto RD, Hazin MAA, Cassão BC, Tedesco-Silva H, Pestana JM (2025). The association between the Kidney Donor Profile Index and
one-year outcomes in Brazilian kidney transplant recipients of standard
criteria donors. J Bras Nefrol.

[2] Clayton PA, Lim WH, Wong G, Chadban SJ (2016). Relationship between eGFR Decline and Hard Outcomes after Kidney
Transplants. J Am Soc Nephrol.

[3] Dahmen M, Becker F, Pavenstädt H, Suwelack B, Schütte-Nütgen K, Reuter S (2019). Validation of the Kidney Donor Profile Index (KDPI) to assess a
deceased donor’s kidneys’ outcome in a European cohort. Sci Rep.

[4] Sanders-Pinheiro H, Colugnati FAB, Denhaerynck K, Marsicano EO, Medina JOP, De Geest S (2021). Multilevel correlates of Immunosuppressive Nonadherence in Kidney
Transplant Patients: the Multicenter ADHERE BRAZIL study. Transplantation.

[5] Ko H, Kim HK, Chung C, Han A, Min SK, Ha J (2021). Association between medication adherence and intrapatient
variability in tacrolimus concentration among stable kidney transplant
recipients. Sci Rep.

[6] Nakamura MR, Requião-Moura LR, Gallo RM, Botelho C, Taddeo J, Viana LA (2022). Transition from antigenemia to quantitative nucleic acid
amplification testing in cytomegalovirus-seropositive kidney transplant
recipients receiving preemptive therapy for cytomegalovirus
infection. Sci Rep.

