# Supplementary material for: Reply to the comment on the “Association between the Kidney Donor Profile Index and one-year outcomes in Brazilian kidney transplant recipients of standard criteria donors”
Source: J Bras Nefrol. 2025 Nov 28;47(4):e20250255rp. doi: 10.1590/2175-8239-JBN-2025-0255rpen (PMC12662568; doi:10.1590/2175-8239-JBN-2025-0255rpen)
Supplement: Translation

# Translation: Resposta ao artigo “Associação entre o *Kidney Donor Profile Index* e os desfechos em um ano em receptores de transplante renal com doadores de critério padrão no Brasil”

**DOI:** 10.1590/2175-8239-JBN-2025-0255rppt

**Authors:** Ana Paula Aquino de Morais, Renato Demarchi Foresto, Maria Amélia Aguiar Hazin, Bianca Cristina Cassão, Helio Tedesco-Silva, José Medina-Pestana, Lúcio Requião-Moura

**Affiliations:** 1 Universidade Federal de São Paulo, Departamento de Medicina, São Paulo, SP, Brasil.; 2 Fundação Oswaldo Ramos, Hospital do Rim, São Paulo, SP, Brasil.

Gostaríamos de agradecer a*Parveen* e*Abid* pela leitura atenta e pelos comentários pertinentes ao nosso artigo, que avaliou o*Kidney Donor Profile Index* (KDPI) em receptores brasileiros de transplante renal (RTR) provenientes de doadores de critério padrão^1^. As observações apresentadas levantam considerações metodológicas e clínicas relevantes, que contribuem para o debate atual sobre a avaliação de risco do doador.

Reconhecemos que a sobrevida do enxerto em longo prazo representa um desfecho mais robusto, por se tratar de um resultado concreto em comparação à taxa de filtração glomerular estimada de um ano em RTRs. Contudo, como nosso estudo representou uma etapa inicial na validação do KDPI para doadores brasileiros, o acompanhamento de um ano foi a abordagem mais acessível e viável nesta fase. Não se deve, entretanto, ignorar que a função do enxerto em um ano, ou mesmo a inclinação da TFGe, já foi previamente associada a resultados de longo prazo^2^ e utilizada como desfecho substituto em estudos de validação anteriores^3^. Ainda assim, uma TFGe satisfatória em um ano não garante a função sustentada do enxerto, uma vez que a adesão do paciente à terapia imunossupressora permanece um desafio bem reconhecido pelas equipes de transplante. O estudo multicêntrico ADHERE Brasil relatou uma prevalência de não adesão de 39,7% em RTRs brasileiros^4^. A má adesão está associada à lesão crônica do enxerto e à sua perda por mecanismos como alta variabilidade nas concentrações sanguíneas de tacrolimo e formação de anticorpos específicos do doador, que podem não se manifestar completamente no primeiro ano^5^. Para superar essas limitações, análises de acompanhamento de longo prazo de nossa coorte estão em andamento e serão publicadas oportunamente, a fim de fornecer dados mais abrangentes.

Em relação à modelagem estatística, concordamos que incluir variáveis clinicamente relevantes, independentemente de sua significância univariada, é uma abordagem válida. Estratégias alternativas, como a predefinição de fatores de confusão com base na relevância clínica, também podem fortalecer a validade externa. Em nossa análise, aplicamos o critério convencional de p < 0,10 nos testes univariados, buscando minimizar o risco de sobreajuste do modelo, sem deixar de incluir variáveis clinicamente importantes. Essas variáveis apresentaram associações significativas e independentes com o desfecho primário, reforçando a robustez dos dados.

Durante a maior parte do período do estudo (2013–2017), o teste de antigenemia pp65 foi o método padrão para o monitoramento de CMV em nosso centro, sendo o teste de PCR implementado posteriormente, após meados de 2017^6^. Esta análise de coorte retrospectiva concentrou-se na função e na sobrevida do enxerto como desfechos primários, com eventos relacionados ao CMV incluídos como variáveis para ajuste nas análises. Nakamura et al.^6^ analisaram posteriormente o período de transição entre a antigenemia e o RT-PCR em nosso centro, e nem a incidência de eventos relacionados ao CMV nem o tempo até o diagnóstico diferiram entre os métodos, exceto por uma duração de tratamento mais longa em comparação com a estratégia preemptiva baseada em RT-PCR.

Em conclusão, apesar das limitações inerentes a um estudo de coorte retrospectivo de centro único, nossos achados demonstram que um KDPI elevado está independentemente associado a piores desfechos, mesmo entre transplantes de doadores de critério padrão, e reforçam a utilidade do KDPI como ferramenta de avaliação de risco do doador em nosso contexto.

## Disponibilidade de Dados

Nenhum dado novo foi criado ou analisado neste manuscrito.
